# Remediation of heavy metal contamination of sediments and soils using ligand-coated dense nanoparticles

**DOI:** 10.1371/journal.pone.0239137

**Published:** 2020-09-30

**Authors:** Yuxiong Huang, Arturo A. Keller

**Affiliations:** 1 Bren School of Environmental Science and Management, University of California at Santa Barbara, CA, United States of America; 2 Shenzhen Environmental Science and New Energy Technology Engineering Laboratory, Tsinghua-Berkeley Shenzhen Institute, Tsinghua Shenzhen International Graduate School, Shenzhen, PR China; VIT University, INDIA

## Abstract

Sediment and soil contamination with toxic heavy metals, including cadmium (Cd^2+^) and lead (Pb^2+^), represents a major long-term remediation challenge. Resuspension of contaminated sediments into the water column, or the uptake of toxic metals from top soil, can lead to exposure of aquatic or terrestrial organisms, followed by bioconcentration, bioaccumulation and biomagnification, which may pose a threat to public health. We have developed a novel nanoscale engineered material, namely ligand-coated dense nanoparticles (Ligand DNPs), which contain a dense WO_3_ nanoparticle core and a shell functionalized with a metal-binding organic ligand (EDTA), to effectively sequester heavy metal ions deeper into the soil and sediments. We demonstrate that one application of Ligand DNPs can remove from 60% to almost 80% of the Cd and Pb in two different soil matrices, driving these metal ions deeper into the sediment or soil column via gravity, and making them less bioavailable. Ligand DNPs can provide a relatively fast, convenient, and efficient *in-situ* approach for the remediation of sediments and soils contaminated with heavy metals.

## Introduction

Heavy metal contamination, such as cadmium (Cd) and lead (Pb), in various environmental media (*e*.*g*. soil, sediments, water) poses a severe threat to ecological and human health as long as they are bioavailable [1,2]. Although there are natural sources of these elements, anthropogenic releases from activities such as metal mining and smelting [3–5], coal combustion [6], trace levels in fertilizers [7,8] and even some wastewater sludge and biosolids [9], can increase concentrations to high levels in soils and sediment beds of lakes and rivers. These toxic elements can be bioavailable to terrestrial and aquatic organisms [10,11], including crop plants (*e*.*g*. rice, wheat) [12,13], and could be further bioaccumulated via the food chain causing damage to humans. Since these metals cannot be degraded, current remediation approaches include excavation or capping, with a very high cost and damage to ecosystems. In many cases, these options are not economically feasible, when the contamination is very wide-spread as is the case of many contaminated farmlands and river beds.

Compared to *ex-situ* remediation technologies, *in-situ* decontamination does not require excavation and transport of contaminated sediment and soil to off-site treatment or disposal facilities, thus it is generally a more practical and economical approach [14]. Conventional *in-situ* soil remediation technologies used for industrial sites contaminated with heavy metals include soil washing/flushing [15,16], chemical immobilization [17,18], electro kinetic extraction [19,20], and phytoremediation [21]. While these technologies may be appropriate for small scale (<1 ha) remediation, they quickly become cost-prohibitive at larger scales. The cost of phytoremediation does not increase much with scale, but the accumulation of metals in the plants presents ecological risks and an eventual disposal cost. Capping sediments essentially destroys habitat [22,23], and the capping may be removed during a large storm event, re-exposing the contaminated media. Thus, there is an urgent need to find better methods to sequester heavy metals to reduce human and ecological risk and ensure better food security.

Chelating agents, for instance, ethylenediaminetetraacetic acid (EDTA), are widely used as extractive agents for heavy metals decontamination [24,25]. Due to its strong metal chelating ability and low cost, EDTA has been used as a metal extraction agent in soil washing [26,27]. However, soil washing can result in unintended mobilization of metals and other pollutants that can be more easily transported by groundwater, and EDTA itself can pose issues as secondary pollution [28]. Thus, a suitable supporting material for EDTA and other chelating agents would minimize the potential unintended environmental implications.

Previously we developed super-paramagnetic EDTA-functionalized nanoparticle adsorbents for water treatment, which were shown to remove a wide range of metal ions with high sorption capacity [29–31]. To date, most nanoscale adsorbents have been applied to the decontamination of aquatic systems [32,33], while very few studies have investigated sediment and soil remediation [34,35]. We have also demonstrated that nanoparticles can readily transport vertically into deeper soil, driven by gravity [36,37]. Thus, we set out to develop a new type of high density nanoscale adsorbent, which can remove heavy metal ions during its downward transport, significantly reducing their bioavailability.

For this study, we selected tungsten oxide (WO_3_) nanoparticles (NPs) as the dense core, which is a relatively low-cost material with high density and low ecotoxicity, to develop the dense nanocomposites that can transport vertically through the porous medium. We first report on the synthesis of EDTA-based Ligand DNPs. We then demonstrate the sorption capacity of Ligand DNPs for Cd^2+^ and Pb^2+^. Next, we evaluate the removal efficiency of Ligand DNPs for Cd^2+^ and Pb^2+^ in two different natural porous matrices. Finally, we report on the *in-situ* remediation performance of Ligand DNPs for Cd^2+^ and Pb^2+^ during gravity-driven vertical transport in these media. The results demonstrate that Ligand DNPs can be applied for effective *in-situ* metal decontamination from soils and sediments.

## Materials and methods

### Chemicals

Tungsten oxide (WO_3_, orthorhombic crystal) nanoparticles (spherical, 23–65 nm in diameter, and 99.95% purity) were purchased from US Research Nanomaterials (USA). Pyridine and toluene were purchased from Alfa Aesar (USA). (3-aminopropyl)triethoxysilane (APTES, 99%) was purchased from Sigma-Aldrich (USA). Cadmium chloride anhydrous, lead chloride, ethylenediaminetetraacetic acid (EDTA), and tris (hydroxymethyl)aminomethane were purchased from Fisher Scientific (USA). Diethyl ether and sodium dihydrogen phosphate were purchased from Acros Organics (Geel, Belgium). Standard Suwannee River natural organic matter (NOM) was obtained from the International Humic Substances Society (IHSS, USA). A NOM stock solution (100 mg/L) was prepared by mixing a known amount of NOM with DI water for 24 h. The pH of the stock solutions was adjusted to 8 with 0.1 M and 0.01 M NaOH and HCl. All chemicals were used as received, without further purification. All solutions were prepared with deionized water (18 MΩ-cm) from a Barnstead NANOpure Diamond water purification system (USA).

### Synthesis of Ligand DNPs

Similar to our previous synthesis strategies [29,31], the core-shell Ligand DNPs were prepared in two steps. The WO_3_ nanoparticles were coated with APTES to form a silane polymer layer via hydrolysis reaction [38]. Then, the surface was modified with EDTA by forming the amide bonds between the EDTA’s carboxylic acid groups and APTES coating’s amino groups [39].

WO_3_ nanoparticles (1.0 g) were dispersed into 40 mL toluene in a flask. After mixing well, 0.4 mL APTES was added to attach an amino group to the WO_3_ particles. Then the flask was connected to a reflux system (WU-28615-06, Cole-Parmer, USA), which was then rotated at 30 rpm (revolutions/minute) in a water bath at 90°C, and refluxed for 2 h. After the solution cooled to room temperature (22°C), 2 mM EDTA and 60 mL pyridine were added. The mixture was again rotated at 30 rpm in a water bath at 90°C in the reflux system for 2 h. After the solution cooled down to room temperature, 100 mL sodium bicarbonate (0.5 M/L) was added to adjust pH to 8.0. Deionized (DI) water was used to rinse the particles twice and then decanted. The same rinsing procedure was performed twice with ethanol and then diethyl ether. The particles were dried at room temperature for 24 h, and stored in a capped bottle prior to use.

### Characterization of Ligand DNPs

Transmission electron microscopy (TEM) images were obtained using a JEOL 1230 Transmission Electron Microscope operated at 80 kV. Scanning electron microscopy (SEM) studies were performed on a FEI XL40 Sirion FEG Digital Scanning Microscope. The surface area and pore volume of Ligand DNPs were determined using a Micromeritics 3Flex Porosimeter. The functional groups of the Ligand DNPs were detected using a Fourier transform infrared (FTIR) spectrometer on a Nicolet iS 10 FT-IR Spectrometer.

### Soil collection and contaminated soil preparation

Two representative soils were used in this study, as examples of the application of Ligand DNPs to treat contaminated porous media. A grassland soil was collected from a flat, well-drained grassy area at the Sedgwick Reserve in Santa Ynez, CA (N 34° 40’ 33.9”, W 120° 02’ 07.6”), and farmland soil was collected from a fallow field at an organic farm in Carpinteria, CA (N 34° 23’ 34.5”, W 119° 28’ 46.9”). The permit for collecting soil samples was authorized by Brenda Juarez. Soil properties can be found in the Supporting Information (SI), in S1 Table in S1 File. Soils were air dried, sieved through a 2 mm mesh, and stored at 4°C until use. The physicochemical properties of the sieved soil samples, including pH, texture, saturation percent, soluble salts, cation exchange capacity (CEC), conductivity, organic content, bulk density, and exchangeable NH_4_, NO_3_, K, and PO_4_, were characterized in our previous study, and available in the SI, shown as S1 Table in S1 File. Total W, Cd, and Pb concentrations of each soil were measured by digesting ~0.3 g soil samples in 10 mL 1:3 HNO_3_: HCl at 200°C for 1.5 h in a microwave digestion system (Multiwave Eco, Anton Paar), followed by analysis via inductively coupled plasma mass spectroscopy (ICP-MS, 7900 Agilent Technology, Santa Clara, CA).

In order to simulate Cd or Pb contamination, 20 g of each type of soil were placed in 50 mL conical test tubes, mixed with 40 mL of 10 mg/L Cd^2+^ or Pb^2+^ solution on an end-over-end shaker (Dayton-6Z412A Parallel Shaft roller mixer, USA) with a speed of 70 rpm at room temperature for 7 days to ensure sufficient equilibration time. Then, the tubes were centrifuged at 10,000 rpm for 20 min to separate soil and the residual Cd^2+^ or Pb^2+^ solution, and the supernatant was collected for residual Cd^2+^ or Pb^2+^ concentration determination by ICP-MS. Soil saturated with Cd^2+^ or Pb^2+^ was preserved at 4°C for the sorption studies. Air dried soil saturated with Cd^2+^ or Pb^2+^ was digested with 1:3 HNO_3_:HCl at 200°C for 1.5 h in a microwave digestion system, then analyzed via ICP-MS to determine the total Cd or Pb content.

### Batch sorption of Cd^2+^ and Pb^2+^

For batch sorption experiments, 20.0 mg of Ligand DNPs were first dispersed in 5 mL DI water, then mixed with 10 g of Cd^2+^ or Pb^2+^ contaminated soil (of each type), in 50 mL conical tubes at pH = 7. Then, these tubes were mixed on the end-over-end system with a speed of 70 rpm at room temperature for 7 days, to ensure sufficient equilibration time.

Adsorption kinetics studies were carried out at the previously stated conditions but for a set amount of time, varying from 6-h, to 12-h, 24-h, 2-day, 3-day, and 7-day. The dosage of Ligand DNPs ranged from 3, to 5, 10, 15 and 20 mg to study the adsorption isotherms at pH 7. To evaluate the potential effect of NOM on the remediation performance of Ligand DNPs, the adsorption isotherms were conducted by first dispersing 3, 5, 10, 15 or 20 mg of Ligand DNPs in 5 mL NOM solution (20 mg/L), then mixing with 10 g of each type of contaminated saturated soil for 7 days.

After mixing the Ligand DNPs with contaminated saturated soil for the specified time, the supernatant and soil were separated by centrifugation. Due to the high density, the immobilized heavy metals adsorbed by Ligand DNPs would be spun down. The treated soil was collected from the top layer to avoid the possible heavy metal binding Ligand DNPs, and then dried in an oven at 60°C for 72 h, then digested for total Cd or Pb content analysis via ICP-MS. All experiments were conducted at ambient temperature (22–25°C).

### Gravity-driven transport through contaminated saturated soils

To investigate the decontamination capability of Ligand DNPs during gravity-driven transport through soil saturated with Cd^2+^ or Pb^2+^, first the contaminated soil was packed into 15 mL conical tubes (17 × 120 mm). An opening with a diameter of 2 cm was made at the bottom of the tubes as the outlet of the system. Suspensions of 20, 40, 60, 80 and 100 mg of Ligand DNPs were dispersed in 5 mL DI water, respectively, and then evenly applied onto the top of each conical tube. After applying the Ligand DNP suspension and allowing the suspension to drip out, the soil columns were placed in horizontal position and air dried overnight then oven-dried at 60°C for 72 h. The dried soil was carefully removed from the conical tube in 3 cm segments, labeled top, middle, and bottom section. Sub-samples (∼0.3 g) were weighed, then digested for Cd or Pb content analysis.

## Results and discussion

### Characteristics of Ligand DNPs

**Fig 1:**
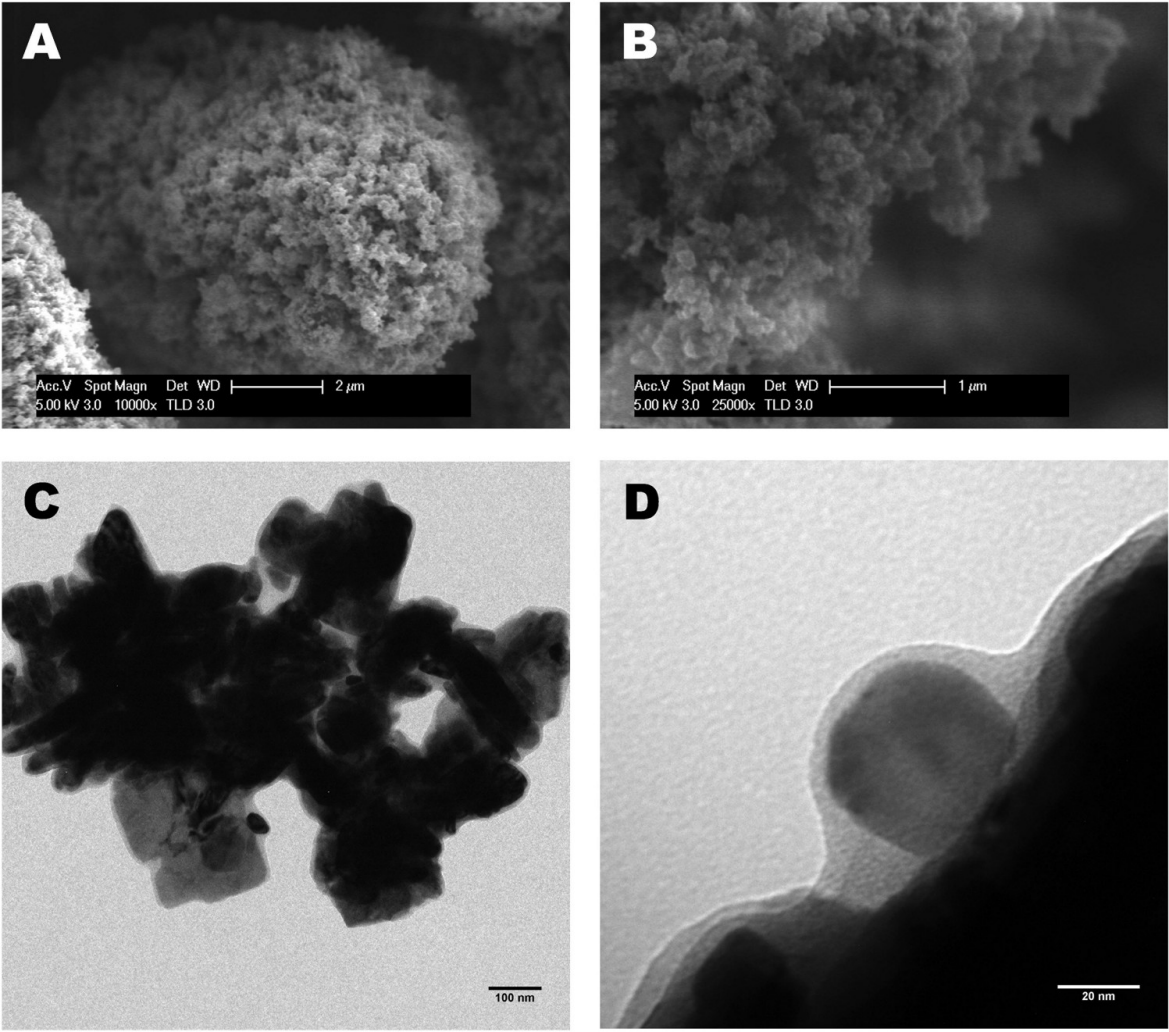
(A) SEM micrographs of Ligand DNPs at 10,000×, scale bar = 2 μm and (B) at 25,000×, scale bar = 1 μm; (C) TEM micrographs of Ligand DNPs with scale bar = 100 nm and (D) scale bar = 20 nm.

Ligand DNPs exhibited a porous surface morphology in the SEM micrograph (Fig 1A and 1B), and the BET surface area was reported as 21.36 m^2^/g with a pore volume of 0.365 cm^3^/g. The surface modification effectively enhanced the surface area of Ligand DNPs, as the pristine WO_3_ nanoparticles had a surface area of 7.61 m^2^/g; this is beneficial for sorption capacity. The core-shell structure can be seen in the TEM images (Fig 1C and 1D), with ~20 nm thick shell layer, as shown in Fig 1D. The primary particle size was around 40–100 nm, but agglomerates or particle clusters were also formed with a size from ~1 to 10 μm. The EDTA was confined on the Ligand DNPs via covalent amide bond between the carboxylic groups of EDTA and amino groups from APTES coating [29]. The FTIR spectra of Ligand DNPs (S1 Fig in S1 File) indicated that Ligand DNPs presented peaks for C = O, N-H, C-N and C-NH2, which were attributed by the APTES coating layer functionalized by EDTA. Determined by the thermal gravity analysis, the mass percentage of EDTA coated onto the Ligand DNPs was around 9.3%. The stability of Ligand DNPs was evaluated in the soil-water system over a 7-day period. Compared to pristine WO_3_ nanoparticles, with the APTES coating, a very limited amount of dissolved W ions (<100 ppb) were released.

The surface charge of Ligand DNPs was determined as negative (-25 to -45 mV) in the pH range of 5 to 8, which helps to prevent aggregation between Ligand DNPs and typically negatively charged soil/sediment particles, and promotes gravity-driven transport of Ligand DNPs through the soil system, as the repulsive forces keep the Ligand DNPs in the central streamlines [40–48]. The density of Ligand DNPs was measured as 7.06 g/cm^3^, which is much higher than the soil particle density (0.981~1.101 g/cm^3^), as shown in S1 Table in S1 File. The higher density provides a larger gravitational driving force for Ligand DNPs to penetrate into the soil and travel further.

### Batch isothermal sorption of Cd and Pb

**Fig 2:**
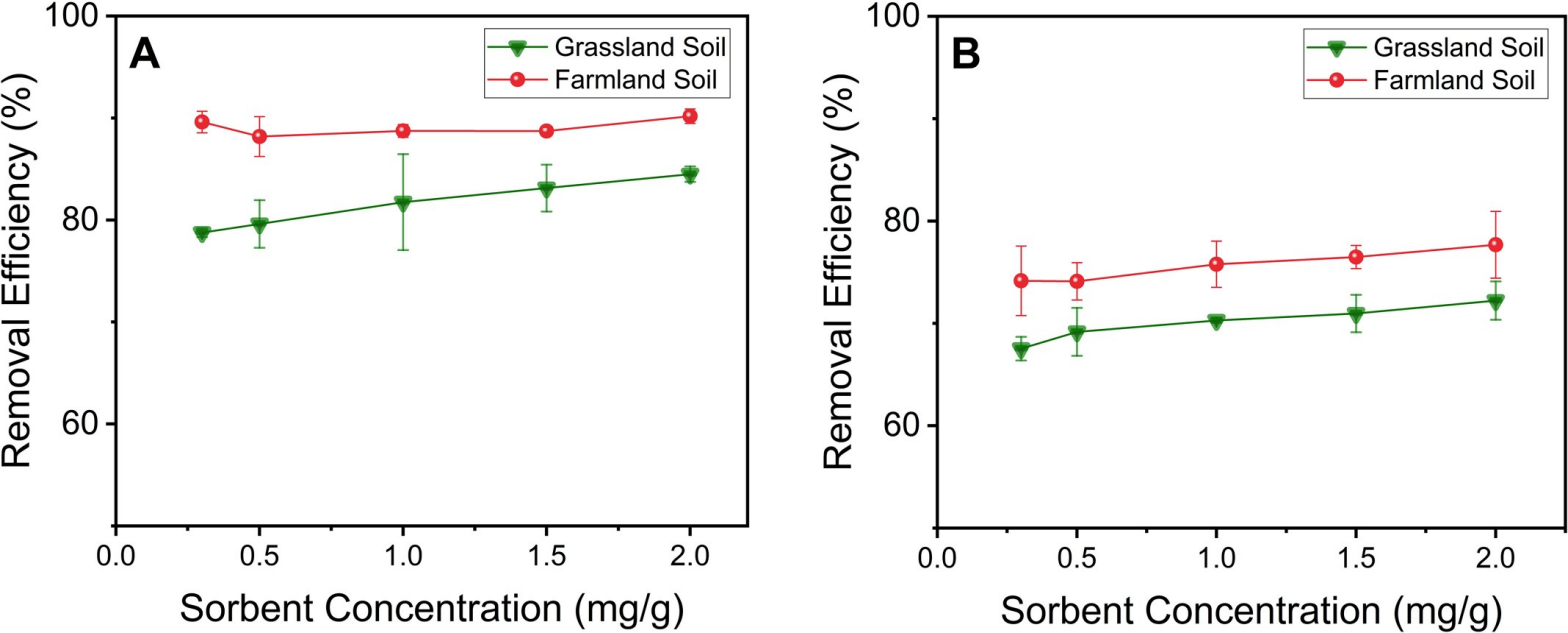
Removal efficiency of Cd^2+^ or Pb^2+^ by Ligand DNPs at different sorbent concentrations (mg sorbent/g soil), in a 7-day batch study at pH 7, from two soil types contaminated with (A) Pb^2+^or (B) Cd^2+^.

The Ligand DNPs were mixed with the two Cd or Pb contaminated soils at pH 7 for 7 days to evaluate their isothermal sorption performance. As shown in Fig 2, the removal efficiency gradually increased as the dosage of Ligand DNPs increased, since this increases the number of active sites. The Ligand DNPs exhibited higher Cd or Pb removal efficiency when applied to farmland soil compared to grassland soil (Fig 2). As shown in S1 Table in S1 File, grassland and farmland soil exhibited significantly different physicochemical characteristics, particularly the organic and ionic concentrations. The CEC (S1 Table in S1 File) of grassland soil (25.8 meq/100g) is considerably higher than the CEC of farmland soil (8.7 meq/100g), which results in higher retention of cations, including Cd^2+^ and Pb^2+^, leading to much lower desorption from the contaminated soil to the soil-water interface. In addition, as shown in S1 Table in S1 File, the electrical conductivity was 142.1 μm/cm for farmland soil and 18.9 μm/cm for grassland soil, indicating a higher concentration of ions (including metal cations) in the leachate of farmland soil compared to grassland soil. Thus, there can be a higher soil-water interface concentration of Cd^2+^ or Pb^2+^ in farmland soil compared to grassland soil, which increases the accessibility and interaction between the active sites of Ligand DNPs and Cd^2+^ or Pb^2+^. In both Cd^2+^ and Pb^2+^ contaminated soil remediation scenarios, Ligand DNPs achieved higher removal efficiencies on contaminated farmland soil than grassland soil (Fig 2).

Ligand DNPs exhibited higher removal efficiencies of Pb^2+^ from both farmland and grassland soils compared to Cd^2+^, which agrees with the sequence of their EDTA complex formation constants (log K, 25°C): 18.04 for Pb^2+^ and 16.46 for Cd^2+^ [49]. It suggests that the complexation between Pb^2+^ or Cd^2+^ and the EDTA-functionalized surface is the dominant removal mechanism [31].

### Kinetics of Cd and Pb removal by Ligand DNPs

**Fig 3:**
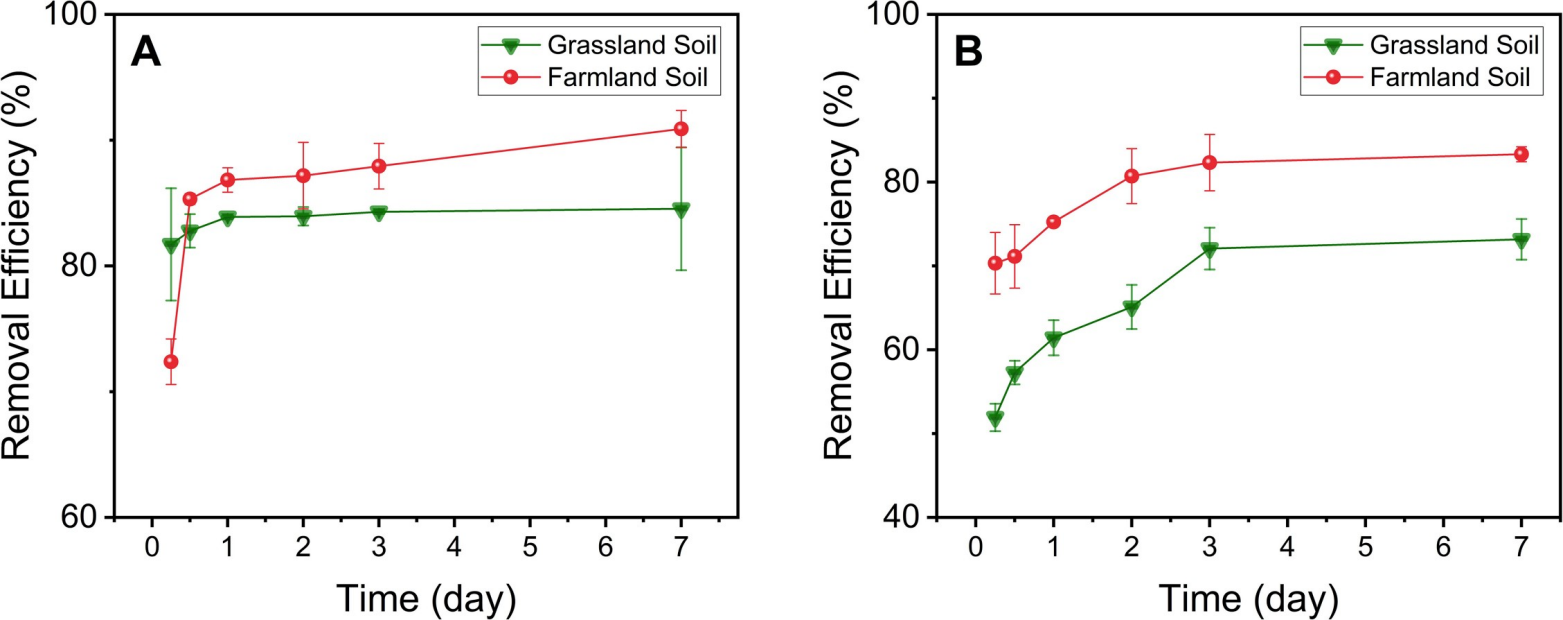
Time-dependent removal of (A) Pb^2+^ and (B) Cd^2+^ by Ligand DNPs at pH 7 and 2 mg sorbent/g soil.

The time-dependent removal of Pb^2+^ or Cd^2+^ by Ligand DNPs in contaminated soil was evaluated in batch studies, as shown in Fig 3. Ligand DNPs showed quick removal of Pb^2+^ in contaminated farmland soils, with over 75% of maximum removal efficiency achieved in the first 6 hours, and a minor increase from 1 to 7 days, when Pb^2+^ in contaminated grassland soils were treated with Ligand DNPs (Fig 3A). Thus, the sorption equilibrium of bioavailable Pb^2+^ with Ligand DNPs can be rapidly reached within 1–2 days, with mixing, in both farmland and grassland soils. Similar removal performance was observed when applying Ligand DNPs for Cd^2+^ soil remediation, as over 70% of the maximum removal efficiency could be achieved in the first 6 hours for both soils (Fig 3B). However, it took up to 3 days of mixing to achieve Cd^2+^ sorption equilibrium (Fig 3B), suggesting Ligand DNPs exhibit a faster removal rate for Pb^2+^ than Cd^2+^, which is due to the stronger binding constant with EDTA [31].

### Effect of NOM on removal efficiency of Cd and Pb

**Fig 4:**
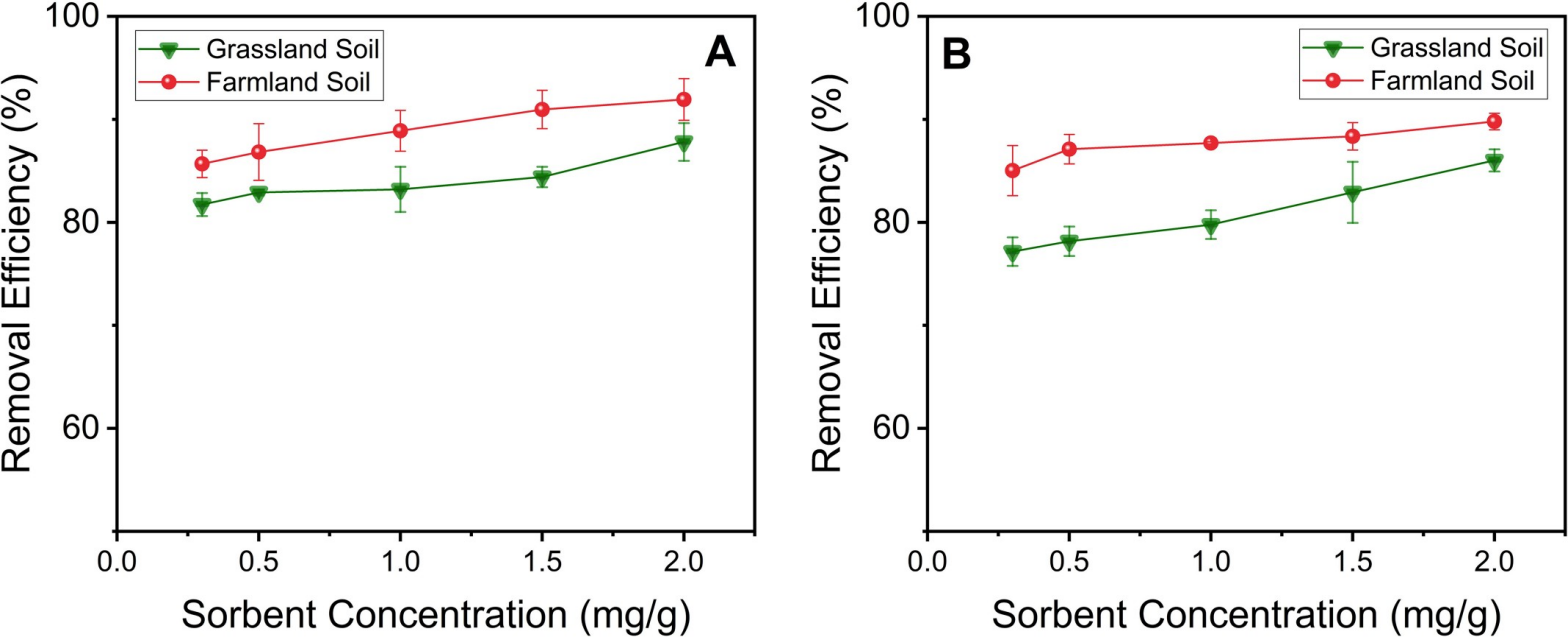
Removal efficiencies of (A) Pb^2+^ and (B) Cd^2+^ by Ligand DNPs at pH 7 in the presence of additional (1%) soluble NOM.

NOM concentration in the soil typically ranges from 0.5% to 5% [50]. In the current study, the original grassland soil had a higher organic content (3.11 ± 0.07%) than the farmland soil (1.44 ± 0.04%), showing a relatively wide range of organic content. In addition, soluble NOM can interfere with, or compete for, metal cation sorption. In order to evaluate the effect of soluble NOM on the removal of Pb^2+^ or Cd^2+^ using Ligand DNPs, an extra 1% NOM was spiked into the Pb^2+^ or Cd^2+^ contaminated soils. Even in the presence of extra soluble NOM, the removal of Pb^2+^ (Fig 4A) using Ligand DNPs did not exhibit significant differences compared to the original soil conditions (Fig 2), while the removal of Cd^2+^ (Fig 4B) actually increased in the presence of NOM, since a significant amount of polar groups (e.g. carboxylic groups) on NOM [51] can also complex Cd^2+^ [52].

### Removal of Cd and Pb during gravity-driven vertical transport

**Fig 5:**
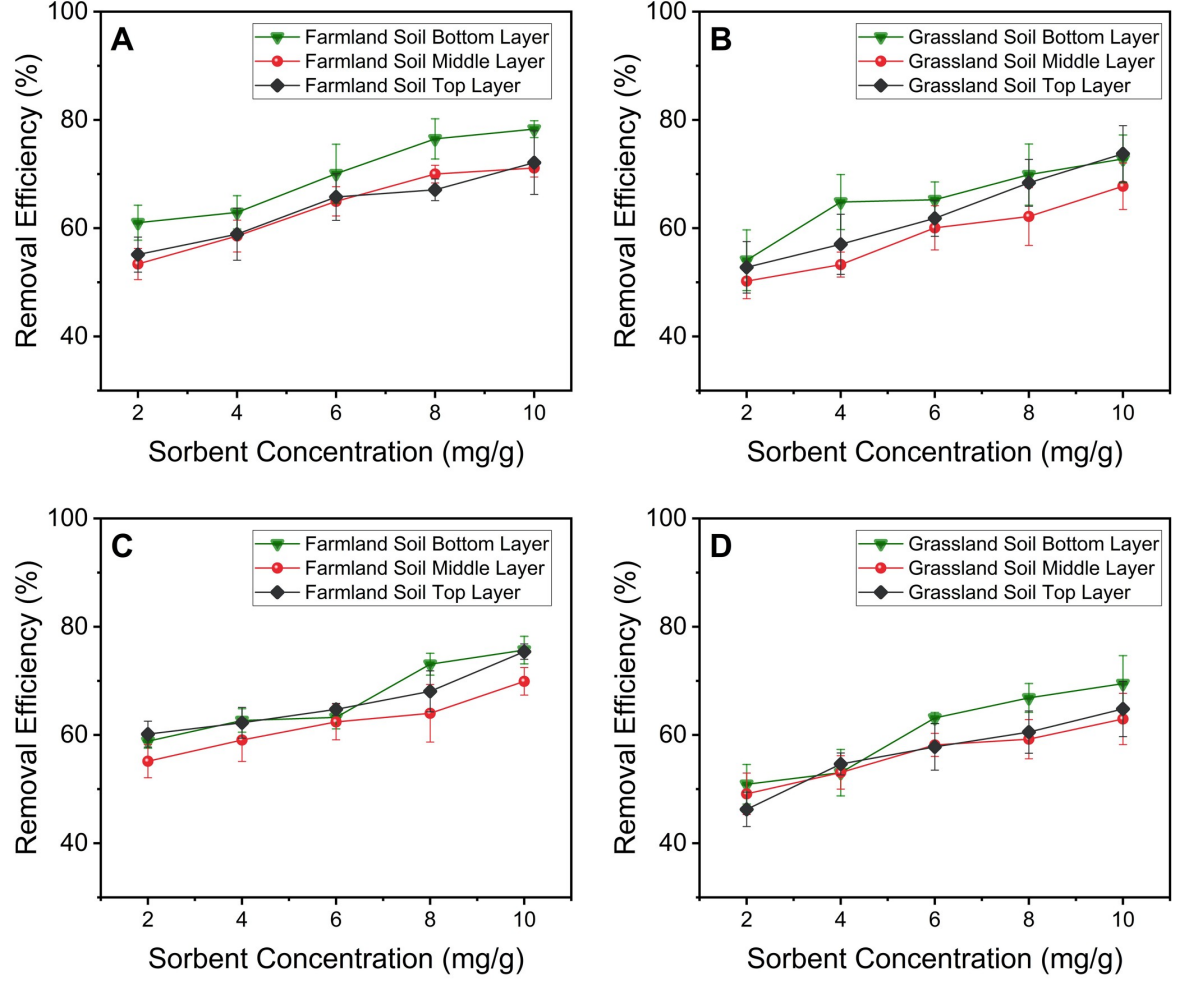
Removal efficiencies using Ligand DNPs for (A) Pb^2+^ contaminated farmland soil; (B) Pb^2+^ contaminated grassland soil; (C) Cd^2+^ contaminated farmland soil; (B) Cd^2+^ contaminated grassland soil. The data was collected across different vertical soil layers.

With a density of 7.06 g/cm^3^, the Ligand DNPs can transport vertically relatively rapidly through the soil, driven by gravity. During their vertical transport, Ligand DNPs can effectively remove Cd^2+^ or Pb^2+^ (Fig 5). In both farmland and grassland soil, the Ligand DNPs could generally pass through the entire depth of the soil column (~ 9 cm) over a 7-day remediation period, with similar removal efficiencies achieved across the vertical layers. Similar to the results of the batch studies, the removal efficiency was better for farmland soil compared to grassland soil, likely due to their differences in cation exchange efficiencies, and removal of Pb^2+^ by Ligand DNPs was slightly higher than Cd^2+^ removal. In addition, while to top and middle layers exhibited similar removal efficiencies, the removal was highest in the bottom layer, where the Ligand DNPs are likely to reside slightly longer, before exiting through the small aperture in the conical tube.

## Discussion

We have demonstrated that Ligand DNPs can readily adsorb heavy metal ions such as Cd^2+^ and Pb^2+^ from contaminated soils. The technology should also be applicable to contaminated sediments in river beds and lakes. Further work would need to be done to demonstrate their applicability to estuaries and coastal areas, where the high ionic strength of those aqueous systems may result in too much attachment of the Ligand DNPs to immobile sediment particles. Further studies are also needed to determine the effect of high Ca^2+^ or Mg^2+^ in the porous medium, but our previous work with Mag-Ligands indicated that this is likely not a concern, given the much higher affinity of EDTA for Cd^2+^ and Pb^2+^ [29]. Ligand DNPs may also heteroaggregate with small (< 1 μm) clay particles, which may still be mobile, resulting in some horizontal as well as some vertical transport; this requires further study.

Porous media properties, in particular the CEC, were shown to influence the ability of the Ligand DNPs to remove the heavy metal ions. However, even Cd^2+^ and Pb^2+^ sorbed onto a soil with a higher CEC were removed by more than 60% in a single treatment, which may be sufficient in many cases to significantly reduce the bioavailability of these metal ions. The presence of organic matter, both that naturally present in the soils and introduced as a more soluble NOM, had only a minor effect on metal ion removal. It is possible that the soluble NOM is also adsorbed onto the Ligand DNPs and thus metal ions sorbed onto NOM are also transported downward; future work could evaluate such interactions between NOM and the Ligand DNPs.

At this stage, the technology is at an early proof-of-concept stage. While one approach would be to attempt to recover the ligand-coated nanoparticles, it is very challenging to do so in a real soil or sediment situation. Thus, we developed this technology to drive these non-degradable contaminants out of a bioavailable zone. This approach could also be used with low levels of persistent organic pollutants, such as PCBs and chlorinated pesticides, which may be near or above a toxicity threshold. The technology is not likely to be cost-competitive for heavily contaminated sites, or it may require many applications of Ligand DNPs to achieve remediation goals. Further work is needed to determine the range of applicability as well as to be tested in more complicated soil system under realistic conditions (e.g., with microbes and plants present).

By using a relatively low-cost material (WO_3_) which itself poses low human and ecological risks, we sought to minimize economic and environmental implications. However, ecotoxicological testing will be needed to establish the dosing of Ligand DNPs that can be safely applied to a contaminated farm or a river bed. Field studies will be needed to determine the feasibility of this approach for this vexing problem.

## Conclusions

Ligand DNPs, with a dense WO_3_ core and an EDTA functionalized porous structured shell layer, were successfully synthesized and evaluated with regards to their removal performance of Cd^2+^ and Pb^2+^ from contaminated soil. The results support our hypothesis that the complexation between metal ions (Cd^2+^ or Pb^2+^) and EDTA was the dominant remediation mechanism, with high removal efficiency (>60 to 80%) for soil decontamination, even with a single application at a dose of 10 mg adsorbent/g soil. Additional doses may result in remediation down to desired clean-up goals. The dense core enables the Ligand DNPs to transport vertically solely by gravity, at a rate that allows adsorption of the heavy metal ions from the porous matrix. Since most of the removal occurs within a few hours of application, the Ligand DNPs are capable of adsorbing the most bioavailable metal ions. Thus, Ligand DNPs are likely to provide a fast, convenient, relatively low-cost and efficient removal approach for sediments and soil contaminated with heavy metals.

## Supporting information

S1 Graphical abstract (TIFF)

S1 File (DOC)
