# Supplementary material for: Remediation of heavy metal contamination of sediments and soils using ligand-coated dense nanoparticles
Source: PLoS One. 2020 Sep 30;15(9):e0239137. doi: 10.1371/journal.pone.0239137 (PMC7526897; doi:10.1371/journal.pone.0239137)
Supplement: S1 File — (DOC) [file pone.0239137.s002.doc]

Supporting information

for

Remediation of Heavy Metal Contamination of Sediments and Soils Using Ligand-Coated Dense Nanoparticles

Yuxiong Huang^1,2^ and Arturo A. Keller^1,*^

^1^Bren School of Environmental Science and Management, University of California at Santa Barbara, CA, USA 93106

^2^Shenzhen Environmental Science and New Energy Technology Engineering Laboratory, Tsinghua-Berkeley Shenzhen Institute, Tsinghua Shenzhen International Graduate School, Shenzhen, 518055, PR China.

**Corresponding author*. Tel: +1 805 893 7548; fax: +1 805 893 7612. Email address: [keller@bren.ucsb.edu](mailto:keller@bren.ucsb.edu)

**S1. Properties of Original Soil Samples**

**Table S1.**  Physicochemical Properties of soils

| **Property** | **Grass soil** | **Farm soil** |
| --- | --- | --- |
| pH ^*^ | 5.90 ± 0.04 | 6.86 ± 0.02 |
| Electrical conductivity (μS cm^−1^) ^*^ | 18.9 ± 0.6 | 142.1 ± 5.4 |
| Cation exchange capacity (meq 100 g^−1^) ^*^ | 25.8 ± 0.1 | 8.7 ± 0.1 |
| Loss-on-ignition organic matter (%)^*^ | 3.11 ± 0.07 | 1.44 ± 0.04 |
| Bulk density (g cm^−3^) ^*^ | 0.981 ± 0.017 | 1.101 ± 0.003 |
| Sand/Silt/Clay (%)^*^ | 54.0/29.0/17.0 | 66.0/22.0/12.0 |
| Saturation percent (%)^*^ | 43.0 ± 0.7 | 28.0 |
| Water content of air-dry soil (wt. %) ^*^ | 10.54 ± 0.02 | 6.23 ± 0.04 |
| Exchangeable PO_4_-P (μg g^−1^) ^*^ | 15.3 ± 0.6 | 51.3 ± 3.0 |
| Exchangeable NH_4_-N (μg g^−1^) ^*^ | 1.28 ± 0.04 | 1.69 ± 0.10 |
| Exchangeable NO_3_-N (μg g^−1^) ^*^ | 11.5 ± 0.5 | 51.9 ± 0.7 |
| Exchangeable K (μg g^−1^) ^*^ | 206 ± 1 | 278 ± 1 |
| Total W (μg g^−1^) | 121.865 ± 39.075 | 111.881 ± 16.165 |
| Total Cd (μg g^−1^) | 0.303 ± 0.022 | 0.066 ± 0.003 |
| Total Pb (μg g^−1^) | ND^†^ | ND^†^ |

^*^ Data was first published in Conway, J. R.; Keller, A. A., Gravity-driven transport of three engineered nanomaterials in unsaturated soils and their effects on soil pH and nutrient release. *Water Research* **2016,** *98*, 250-260.

^†^ND refers to under analytical instrument detect limit.

*
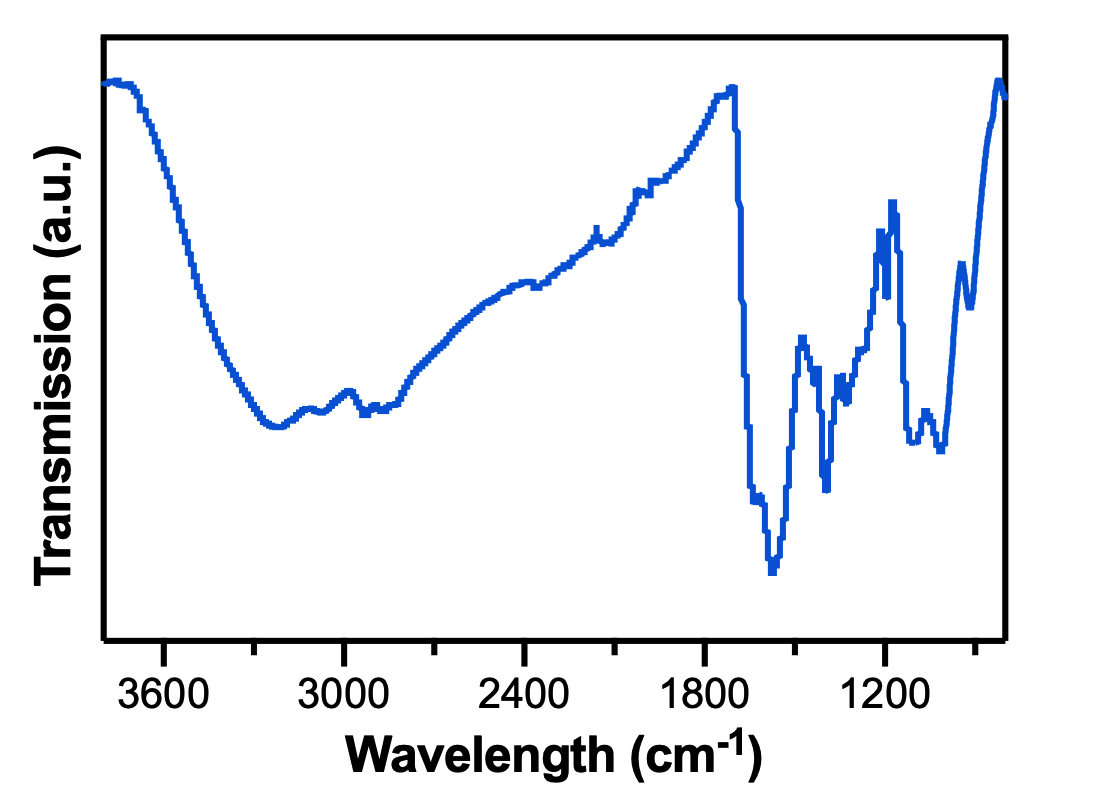
*

**Figure S1.** FTIR spectra of Ligand DNPs
